# The Oxidative Stress Imbalance in Children and Adults with IBD and Associated Factors

**DOI:** 10.3390/nu18091458

**Published:** 2026-05-01

**Authors:** Sara Quattrini, Tiziana Galeazzi, Chiara Monachesi, Alessandra Palpacelli, Giulia Catassi, Claudia Quatraccioni, Giulia Annulli, Antonio Di Sario, Laura Cianfruglia, Monia Orciani, Tatiana Armeni, Andrea Faragalli, Rosaria Gesuita, Maria Elena Lionetti, Carlo Catassi, Simona Gatti

**Affiliations:** 1 G. Salesi Children’s Hospital, Department of Pediatrics, Polytechnic University of Marche, Via Corridoni 11, 60123 Ancona, Italy; sara.quattrini29@gmail.com (S.Q.); c.catassi@univpm.it (C.C.); 2 Pediatric Gastroenterology Hepatology and Cystic Fibrosis Unit, Fondazione IRCCS Caà Granda, Ospedale Maggiore Policlinico di Milano, 20122 Milan, Italy; 3 Department of Gastroenterology and Transplantation, Polytechnic University of Marche, 60126 Ancona, Italy; 4 Division of Gastroenterology and Digestive Endoscopy, Senigallia Hospital, AST Ancona, 60019 Ancona, Italy; 5 Department of Odontostomatologic and Specialized Clinical Sciences, Polytechnic University of Marche, 60126 Ancona, Italy; 6 Department of Clinical and Molecular Sciences-Histology, Polytechnic University of Marche, 60126 Ancona, Italy; 7 Center of Epidemiology, Biostatistics and Medical Information Technology, Polytechnic University of Marche, 60126 Ancona, Italy

**Keywords:** IBD, oxidative stress, FRAP, antioxidant capacity, inflammation

## Abstract

**Background/Objectives**: An imbalance in oxidative stress (OS) has been implicated in the pathogenesis of Inflammatory Bowel Disease (IBD). We compared OS status in IBD children and adults versus healthy controls by exploring variables impacting the OS disruption in IBD. **Methods**: Total antioxidant capacity (ferric-reducing ability of plasma (FRAP)), reactive species (ROS), oxidative products (advanced oxidation protein products (AOPPs) and thiobarbituric acid reactive substances (TBARSs)), and antioxidant defenses (glutathione, GSH and intracellular activity of the main antioxidant enzymes) were evaluated. Correlations between OS markers, clinical features, disease characteristics, and inflammatory indices were explored. **Results**: Eighty-two IBD patients (67.5% in clinical remission) and 73 healthy subjects were enrolled. IBD children showed significant FRAP reduction compared to controls and IBD adults (*p* < 0.0001), increased AOPPs and reduced GSH compared to controls (*p* < 0.0001 and *p* = 0.0011, respectively), higher total GSH (*p* = 0.020), and lower TBARSs (*p* = 0.023) compared to IBD adults. In the pediatric group, FRAP was significantly reduced in those with IBD and increased in older subjects and males, while AOPP levels were positively affected by increasing age. In the total IBD cohort, higher FRAP was associated with male gender, increasing age, overweight, and mesalazine therapy. The diagnosis of Ulcerative Colitis was associated with lower FRAP and AOPP levels compared to Crohn’s disease. Increased fecal calprotectin significantly decreased the total antioxidant capacity. **Conclusions**: The antioxidant system shows significant differences in IBD compared to controls, particularly in the pediatric group. The observed pediatric–adult pattern may suggest age-related differences in oxidative balance, but these findings should be interpreted with caution, given the modest sample size. **Clinical Trial Registration Number**: NCT04513015.

## 1. Introduction

Inflammatory Bowel Disease (IBD) is a chronic inflammatory intestinal condition that can present at any age with different clinical forms, including Crohn’s Disease (CD), Ulcerative Colitis (UC), and IBD-unclassified (IBD-U). Apart from a tiny proportion of cases that develop IBD as the consequence of a specific genetic defect (monogenic IBD) [1], the vast majority of IBD are multifactorial disorders, related to the disruption between the immune system and the gut microbiota, triggered by several environmental factors, in genetically predisposed individuals [2].

Pathogenesis of IBD is still largely unexplained, and oxidative stress (OS), defined as the result of an imbalance between prooxidant and antioxidant mechanisms, has been implicated in the development and maintenance of inflammation and aberrant immune response in IBD [3]. OS contributes to bowel damage through different mechanisms, including increased inflammatory mediators and direct damage to the intestinal barrier [4,5]. The inflammatory mucosal infiltration in IBD, mediated by activated phagocytic immune cells, generates a prooxidant environment, characterized by the abundance of reactive oxygen (ROS) and nitrogen species (RNS) that damage key macromolecules (lipids, proteins, and nucleic acids) and determine cell injury and increased permeability, perpetuating the ongoing inflammation. These mechanisms are generally counterbalanced by antioxidants, including first-line endogenous enzymes, that prevent and neutralize free radicals such as superoxide dismutase (SOD), catalase (CAT), glutathione peroxidase (GPX), peroxiredoxins, and paraoxonase (PON1), and metal-chelating proteins (transferrin, ceruloplasmin, and albumin), which sequester free iron and copper, preventing them from participating in the Fenton reaction. The second line of antioxidant defense includes free radical scavengers such as glutathione (GSH), uric acid, cysteine, bilirubin, carotenoids, and vitamins A, E, and C, which neutralize free radicals by donating electrons [6,7,8]. The third and fourth lines of defense rely on the depletion of oxidized molecules by prooxidants at the molecular and cellular levels, respectively [9].

Several levels of evidence (including clinical, in vivo, and in vitro studies) have shown an impaired antioxidant capacity with a consequent accumulation of oxidative products in IBD, and results have been recently systematically reviewed and summarized [10,11,12,13]. OS is considered one of the principal triggers of neoplastic evolution in IBD patients [3]. Furthermore, the risk of IBD has been linked to several oxidative stress-relevant genetic loci [14]. Lastly, a therapeutic effect of some antioxidant molecules or dietary compounds is emerging as a potential treatment for IBD [3]. Whether the role of OS impairment is causative in the development and evolution of IBD or is mainly the consequence of the inflammation remains a matter of debate.

Comparative studies have demonstrated remarkable differences in the clinical course and treatment response of IBD in pediatric and adult-onset cases [15,16]. Despite similar pathogenetic mechanisms, age-related factors and exposure to different environmental factors can contribute to heterogeneous clinical scenarios in pediatric and adult IBD [17,18]. Epidemiological data clearly show significant changes in IBD presentation and age profile, with an increasing incidence among younger [19] and older populations [20,21]. Pediatric and adult subjects are potentially exposed to a different load of pro-oxidative factors, including smoking, dietary factors, environmental agents, and drugs; furthermore, age certainly impacts the antioxidant capacity of humans [22]. The direction of such modifications is less clear in children [23].

Measuring OS markers could facilitate the understanding of IBD pathophysiology and differences in pediatric and adult forms, and may also be useful in defining disease severity. With this study, we aimed to evaluate the OS status in children and adults with IBD compared to healthy controls and to explore the correlation between OS parameters, clinical variables, and the burden of IBD-related inflammation.

## 2. Materials and Methods

### 2.1. Study Design

The OXIBDiet (NCT04513015, approval date: 27 July 2020) was a prospective, multicentric, no-profit study coordinated by the Department of Pediatrics at the Polytechnic University of Marche, Ancona, Italy. Children and adults were enrolled at the Department of Pediatrics and the Department of Gastroenterology of the Polytechnic University of Marche, Ancona, Italy, respectively, between September 2020 and December 2023. The study protocol was approved by the Marche Regional Ethical Committee (CERM, protocol number 2019-174, approval date: 30 May 2019), which verified the conformity to the Good Clinical Practices and the Declaration of Helsinki. All patients gave informed consent for participation in the study and for data publication.

### 2.2. Primary and Secondary Outcomes

The primary outcome of the study was the assessment of the serum total antioxidant capacity, measured as ferric-reducing activity of plasma (FRAP) in patients with IBD compared to controls. Secondary outcomes included other oxidative stress markers, including levels of reactive oxygen species (ROS) and glutathione, antioxidant enzyme activities, thiobarbituric acid reactive substances (TBARSs), and advanced oxidation protein products (AOPPs).

### 2.3. Inclusion and Exclusion Criteria

Children and adults (age range: 6–80 years old) with an established diagnosis of IBD (including CD, UC, and IBD-U), with different disease activities, were enrolled at each recruitment center during an outpatient visit or inpatient admission. A group of healthy subjects was enrolled; specifically, healthy adults were enrolled among the medical staff, while healthy children were recruited at the general pediatric department of the same hospital and included subjects coming for a general pediatric check-up. Exclusion criteria were permanent stoma, previous or current diagnosis of cancer, cardiovascular disease, ischemic disease, Alzheimer’s disease, type 2 diabetes, pregnancy, and diseases requiring a specific dietetic treatment (i.e., food allergies, celiac disease, etc.).

### 2.4. Socio-Demographic Data, Disease Characteristics, and Anthropometric Data

At recruitment, clinical evaluation was recorded in the Case Report Form (CRF) and included socio-demographic data (age, gender, ethnicity), lifestyle habits (type of diet, smoking habit), and disease characteristics. Specifically, for IBD patients the following data were collected: type of IBD (CD, UC or IBD-U); phenotype according to the Paris classification for children [24] and Montreal for adults [25]; age at diagnosis; duration of disease; extraintestinal manifestations; current medications (including vitamin supplements and enteral nutrition); timing and type of previous IBD-related surgery.

For IBD children, disease activity was evaluated using the weighted Pediatric Crohn’s Disease Activity Index (w-PCDAI) [26] for CD, and the Pediatric Ulcerative Colitis Activity Index (PUCAI) [27] for UC and IBD-U. Disease activity scores were classified using previously validated cut-off points: remission (w-PCDAI < 12.5 or PUCAI < 10), mildly active disease (w-PCDAI: 12.5–40 or PUCAI: 10–34), moderately active disease (w-PCDAI > 40–57.5 or PUCAI: 35–64) and severely active disease (w-PCDAI > 57.5 or PUCAI: 65–85). For IBD adults, disease activity was evaluated using the Harvey–Bradshaw index [28] for CD and the Mayo score [29] for UC and IBD-U. Disease activity scores were classified using previously validated cut-off points: remission (Harvey–Bradshow < 5 or Mayo score < 2), mildly active disease (Harvey–Bradshaw: 5–7 or Mayo score: 2–4), moderately active disease (Harvey–Bradshaw: 8–16 or Mayo score: 5–7) and severely active disease (Harvey–Bradshaw > 16 or Mayo score > 7).

Anthropometric parameters (weight, height, body mass index (BMI)) were collected by the investigators at recruitment. Weight and height were measured at each center, body mass index (kg/m^2^) was calculated and the z-score of weight, height and BMI were extracted considering the general Italian population as a reference for the pediatric groups [30]. Undernutrition was defined as mild (BMI z-score between −1 and −2), moderate (BMI between −2 and −3) or severe (BMI z-score ≤ −3). A BMI z-score between +1 and +2 was considered “overweight”, a BMI z-score between +2 and +3 was considered “obesity” and a BMI z-score ≥ 3 was considered “severe obesity” [31,32].

### 2.5. Laboratory Parameters, Inflammatory Markers, and Oxidative Stress Biomarkers

#### 2.5.1. Sample Collection and Storage

Fasting blood samples were collected at recruitment in both IBD patients and controls. Blood was centrifuged at 3000 rpm for 10 min; serum was carefully separated and stored at −80 °C until examination (or until the assessment of total antioxidant capacity (FRAP) and macromolecule oxidative products (advanced oxidation protein products, (AOPPs) and thiobarbituric acid reactive substances (TBARSs).

The routine laboratory tests, including complete blood count, ferritin (ng/mL), glucose (mg/dL), albumin (g/dL), ALT (U/L), creatinine (g/dL), lipid profile (total cholesterol, HDL cholesterol, triglycerides, mg/dL), zinc (ug/dL), vitamins A (μg/dL), D (ng/mL), E (μg/dL) and C (mg/L) and total plasma proteins, were performed using automatic standard procedures. C-reactive protein (CRP, mg/dL), erythrocyte sedimentation rate (ESR, mm/h), and fecal calprotectin (FC, mg/kg) were measured as markers of inflammation.

#### 2.5.2. Isolation of Mononuclear Cells

Human peripheral blood mononuclear cells (PBMCs) and plasma were isolated from peripheral blood EDTA samples by density centrifugation using a Leucosep™ tube and Biocoll as separation medium (Greiner Bio-One S.r.l). Briefly, the Leucosep™ tube was filled with separation medium and centrifugated. The dilute (1:1 with PBS) anticoagulated sample was further centrifugated and the enriched cell fraction (PBMCs) was harvested and washed twice with PBS. Both cells and plasma were stored at −80 °C until the analysis.

#### 2.5.3. Ferric-Reducing Activity of Plasma

FRAP values (µmol Fe^2+^) were obtained by measuring ferric to ferrous ion reduction at low pH (0.3 M acetate buffer, pH 3.6), coupled with TPTZ solution (10 mM 2,4,6-tripyridyl-s-triazine (TPTZ) in 40 mM HCl) and 20 mM FeCl_3_·6H_2_O in a volume ratio of 10:1:1, whose absorbance was measured at a wavelength of 593 nm [33].

#### 2.5.4. Advanced Oxidation Protein Products

AOPPs (µmol/g of plasma proteins), expressed in equivalent of chloramine T concentration, were assessed in plasma according to the spectrophotometric method of Witko-Sarsat et al. [34], and corrected to the total plasma protein concentration.

#### 2.5.5. Thiobarbituric Acid Reactive Substances

TBARSs (µM MDA) were evaluated as an index of lipid peroxidation and measured in plasma spectrophotometrically at 532 nm by adopting the thiobarbituric acid-reactive substances (TBARSs) assay kit (Cayman Chemical, Ann Arbor, MI, USA). A linear calibration curve was calculated from differently concentrated pure malondialdehyde (MDA) solutions.

#### 2.5.6. Reactive Oxygen Species

Intracellular ROS levels were detected immediately by flow cytometry using H2DCFDA (C400) as a probe. Cells were suspended at a final concentration of 10^6^ cell/mL in pre-warmed PBS containing 10 μM probe. After incubation for 30 min in the dark at 37 °C, cells were washed twice in PBS and stained with 10 μg/mL propidium iodide (PI). Fluorescence was measured on a Guava Easy Cyteflow cytometer (Merck Millipore, Darmstadt, Germany) using an excitation wavelength of 488 nm. Emissions were recorded using the green channel for carboxy–DCF and the red channel for PI. The cells permeable to PI were excluded from the cell population considered for the ROS production to avoid false negatives.

#### 2.5.7. Total Glutathione

Total glutathione (µM) was measured by a commercially available glutathione assay kit (Cayman Chemical, Ann Arbor, MI, USA, Cat. No. 703002) and used in accordance with the manufacturer’s protocol. The assay utilizes the glutathione reductase (GR) recycling assay in the presence of 5,50-dithiobis (2-nitrobenzoic acid) (DTNB). GSH reacts with DTNB to produce a yellow-colored TNB (5-thio-2-nitrobenzoic acid). The rate of TNB production is directly proportional to the formation of GSH, which is measured at 412 nm using a plate reader.

#### 2.5.8. Antioxidant Enzyme Activity

Superoxide dismutase (SOD), glutathione peroxidase (GPX), glutathione reductase (GR), glutathione S-Transferase (GST), and catalase (CAT) activities (nmol/min/mg of protein) were evaluated in PBMCs using specific kits (Cayman Chemical, Ann Arbor, MI, USA) in accordance with the manufacturer’s instructions. Briefly, SOD activity assay was based on the SOD capacity to cause dismutation of the superoxide radicals generated by xanthine oxidase and hypoxanthine. The absorbance was read at 450 nm. One unit of SOD was defined as the amount of enzyme needed to exhibit 50% dismutation of the superoxide radicals. GPX activity was measured indirectly by a coupled enzyme system with glutathione reductase. NADPH is consumed by glutathione reductase to convert the formed GSSG to its reduced form (GSH). GPX activity was monitored by the decrease in absorbance at 340 nm using cumene hydroperoxide as substrate. GR activity was evaluated by measuring NADPH oxidation. A decrease in absorbance at 340 nm due to NADPH oxidation during GSSG reduction was directly proportional to the GR activity. Total GST activity was measured using the conjugation of 1-chloro-2,4-dinitrobenzene (CDNB) and GSH as substrate and measuring the absorbance of resulting products at 340 nm, according to the method of Habig and colleagues [35]. The method to measure CAT activity is based on its reaction with methanol in the presence of H_2_O_2_, using formaldehyde as standard and -amino-3-hydrazino-5-mercapto-1,2,4-triazole (Purpald reagent) as chromogen [36]. H_2_O_2_ was added to initiate the reaction, and CAT activity was defined as the amount of enzyme that caused formation of 1 nmol of formaldehyde per minute at 25 °C. The absorbance was read at 540 nm with the use of a plate reader.

### 2.6. Statistical Analysis

A descriptive analysis was conducted to compare the main characteristics of subjects in the case and control groups. Medians and interquartile ranges (IQRs), as well as absolute and percentage frequencies, were used to summarize quantitative and qualitative variables, respectively. Differences between groups were assessed using the Wilcoxon rank-sum test for continuous variables and the Chi-square or Fisher’s exact test for categorical variables. The characteristics of children and adults with IBD were also compared.

Quantile regression was employed to assess the impact of subjects’ characteristics and type of disease on the median values of the main OS parameters (FRAP, AOPPs, and SOD), considered as dependent variables. The independent variables included in the quantile regression analysis were gender, age, presence of disease, inflammatory indexes (ESR, CRP, or fecal calprotectin), nutritional status, and serum concentrations of vitamins A, C, D, E, and zinc. Given the modest sample size, the most parsimonious model was identified using a stepwise variable selection procedure. Analyses were performed on available complete cases for the variables included in each model, and no imputation of missing data was applied. Results were reported as point and 95% confidence interval (95% CI) estimates. Regression coefficients were considered not statistically significant if the zero value was included in the interval. The analysis was stratified by age group (children and adults) and additionally in a combined model using a categorical variable to denote adulthood (Adult yes vs. no). In this final model, variables such as the type of disease (UC/IBDU vs. CD), use of drugs (biologics, immunomodulators, and oral mesalazine), fecal calprotectin, and nutritional status were considered as independent variables.

## 3. Results

The overall sample included 75 children (41 IBD and 34 controls, median age: 12.7 years, IQR: 10.8–14.2, 54.6% females) and 80 adults (41 IBD and 39 controls, median age: 31.3 years, IQR: 27–42.7, 53.7% females). The socio-demographic, clinical, and disease characteristics, as well as the nutritional status of the whole population, are summarized in Table 1.

IBD children, in comparison to adults, were more frequently on immunomodulatory therapy (*p* < 0.001). In terms of nutritional status, IBD children were more often underweight, while adults had higher rates of overweight or obesity (*p* = 0.010).

The levels of oxidative stress biomarkers are shown in Table 2.

IBD children showed significantly reduced total antioxidant capacity (FRAP) (*p* < 0.001), higher levels of AOPPs (*p* = 0.011), and a reduced level of GSH (*p* = 0.033) in comparison to controls. The intracellular activity of CAT and SOD was significantly increased (*p* = 0.010 and *p* = 0.039, respectively), compared to controls. Adults with IBD did not show significant differences in terms of oxidative markers in comparison to the control group except for the activity of the CAT enzyme. The distributions of the markers in the two groups of adult patients were essentially comparable, as evidenced by the median values and interquartile ranges; although this finding should be interpreted with caution, as it suggests that the lack of statistical significance may not be attributable to the small number of patients per group.

Comparing IBD children and adults, adults had higher FRAP levels (*p* < 0.001), while children had higher total GSH levels (*p* = 0.020) and lower TBARS levels (*p* = 0.023).

The Supplementary Table S1 reports comparisons of further biochemical parameters, including inflammatory markers and vitamin levels. IBD children showed a raised platelet count (PLT) (*p* = 0.039) and erythrocyte sedimentation rate (ESR) (*p* = 0.032), and exhibited lower albumin levels (*p* = 0.001) compared to controls.

IBD patients (both children and adults) with active disease and remission (based on clinical scores) did not show any difference in FRAP, TBARSs, or ROS. AOPP levels were significantly increased in IBD children with active disease compared to patients in remission, but not in adults (Table 3).

### Quantile Regression Analysis

Table 4, Table 5 and Table 6 summarize the results of the quantile regression analysis for the pediatric cohort.

FRAP was significantly reduced in patients with IBD (median value of FRAP reduced of 53 µmol/equivalent FeSO_4_ (95% CI −64; −42), while increasing age, male gender, and vitamin A serum levels had a positive impact on FRAP levels. AOPP levels were positively affected by increasing age but not by gender or IBD status. Vitamin C showed a significant positive association with both AOPP (median increase of 0.052, 95% CI: 0.024 to 0.258) and SOD (median increase of 0.198, 95% CI: 0.02 to 0.316). Fecal calprotectin, as a disease activity marker, significantly increased the median values of AOPP and SOD, respectively, by 0.002 (95% CI: 0 to 0.005) and 0.003 (95% CI: 0.001 to 0.005).

The results of the quantile regression analysis for the adult group are reported in Supplementary Table S2. The presence of the disease did not significantly impact FRAP and AOPP levels. Age showed a strong positive association with FRAP, with an increase in the median level of FRAP of 47.542 (95% CI: 32.83; 69.623), indicating increased antioxidant capacity with age. There was no significant association between age and AOPP. Age decreased the median level of SOD, suggesting a reduced enzymatic antioxidant activity with increasing age. No significant associations between gender and the oxidative stress markers were observed.

Table 7, Table 8 and Table 9 show the quantile regression analysis for the total IBD cohort (IBD children and adults), adjusted for disease type, treatment exposure, fecal calprotectin, and nutritional status.

Significant associations were observed between patient characteristics and oxidative stress markers (FRAP, AOPPs, and SOD). Higher levels of FRAP were observed in males and adults. Being overweight and receiving 5-ASA therapy was also associated with increased FRAP levels. Conversely, lower FRAP levels were associated with the diagnosis of UC/IBD-U vs. CD and higher fecal calprotectin levels. Significantly lower levels of AOPPs were found in patients with UC/IBD-U compared to those with CD. Higher levels of SOD were associated with the diagnosis of UC/IBD-U compared to CD.

## 4. Discussion

A growing number of studies in recent years have suggested that in IBD, OS is not only a consequence of chronic inflammation but also has a crucial role in the pathogenesis [4]. In this study, performed on a total of 155 subjects (82 IBD and 73 healthy subjects), different markers of pro- and antioxidant activities were assessed in both children and adults with IBD and a control group, and correlations with disease parameters were evaluated.

The major finding of this study is a reduced antioxidant capacity in IBD children compared to healthy children and adults with the same disease. Additionally, IBD children exhibited an accumulation of oxidation protein products compared to controls, and exhibited different activity of several antioxidant enzymes. Conversely, adults with IBD did not show differences in the total antioxidant capacity compared to their controls.

Previous studies, mainly focused on adults with IBD, have described a reduced total antioxidant capacity (evaluated with different methods) in several cohorts, and a recent meta-analysis has reported a significant reduction in the global antioxidant capacity in IBD versus healthy controls, and in patients with active CD versus those with inactive disease [10]. In the three IBD studies that used FRAP as the measure of total antioxidant capacity (TAC), two of them revealed a decreased FRAP in adults with CD compared to controls [37,38] and another detected no differences [39]. We decided to assess the total antioxidant capacity by using FRAP because this is an easy-to-determine and reproducible measure of TAC [33]. In fact, in acidic conditions plasma antioxidant compounds donate electrons, reducing ferric iron (Fe^3+^) to ferrous iron (Fe^2+^), allowing for a reliable estimation of the global antioxidant capacity, as it gathers whole scavenging abilities of plasma. A reduction in FRAP has been demonstrated in many inflammatory and other autoimmune conditions, such as rheumatoid arthritis [40] and type-1 and -2 diabetes [41,42]. In IBD children, a small pediatric study failed to demonstrate any significant alteration in the TAC of IBD subjects [43]. To the best of our knowledge, this is the first controlled study that deeply investigated a pediatric IBD cohort and compared the oxidative imbalance in children and adults with IBD.

Our results were further reinforced by the evaluation of the oxidative damage through the measurement of the advanced oxidation protein products. AOPPs are di-tyrosine-containing and cross-linking products, formed by the reaction of plasma proteins, mainly albumin, with chlorinated compounds resulting from the activity of myeloperoxidase (MPO) [34,44]. AOPPs are recognized as both oxidative protein damage markers and mediators of inflammation. Protein oxidation determines the inactivation of proteins (essential for cellular protection), and enhances intraepithelial reactions that disrupt the integrity of the mucosal barrier [45]. AOPPs have been suggested as one of the most accurate biomarkers of oxidative stress [34]. Specifically in the context of IBD, AOPPs distinguish IBD subjects from healthy controls and patients with active disease from subjects in clinical remission [39,46,47,48,49]. The same differences emerged in our pediatric IBD cohort, but not in adults. In a small pediatric interventional study, AOPPs were significantly increased in CD children with remission status compared to healthy controls [49], and decreased after 5 weeks of polyphenol administration. Our data, although preliminary, suggest AOPPs as a potential marker of pediatric IBD, reflecting the clinical inflammatory burden in IBD children. In both animal and human studies, AOPPs were found to be directly correlated to intestinal epithelial damage and inflammatory changes, with data suggesting a possible pathogenetic role contributing to IBD progression [50]. Furthermore, targeting AOPP-induced cellular mechanisms might emerge as a promising therapeutic option for patients with IBD.

The imbalance in the OS homeostasis in IBD children was further reinforced by the decrease in the GSH, a second-line free radical scavenger, acting also as a cofactor for different antioxidant enzymes. Previous reports comparing IBD subjects to controls reported controversial results. Levels of GSH were found to be elevated among pediatric CD patients [51], while adult studies have reported almost a reduction, especially in subjects with active and/or complicated diseases [37,38,52]. We found lower GSH levels in IBD children compared to healthy peers, but levels were higher compared to IBD adults. Reduced GSH levels, along with increased AOPPs, have been found in UC patients with mild to moderate dysplasia and therefore hold potential as early dysplasia markers [53].

Differences in the antioxidant capacity between IBD children and adults do not seem to be strictly related to discrepancies in disease activity. Although IBD children are generally considered to have more severe disease, in our cohort adults and children were comparable for measures of disease activity, including calprotectin levels. In the univariate analysis, disease activity did not influence levels of FRAP. The only difference was found in the levels of AOPPs, which were elevated in IBD children with active disease compared to children in clinical remission, but not in adults (Table 3).

The second part of our study investigated variables impacting the main oxidative markers (FRAP, AOPPs, and SOD), separately (in children and adults) and in the entire cohort. In the three groups, increasing age had a positive impact on FRAP levels, indicating increased antioxidant capacity with age in subjects with IBD. The product of protein oxidation (AOPPs) was inversely affected by age, with the youngest subjects showing increased protein oxidation. Although this result was obtained using a multiple regression model adjusted for potential confounding factors, additional residual confounding factors not included in the present study may have played a role. These findings seem to be counterintuitive compared to what is generally known about oxidative stress and aging [22,54,55] in the general population; however, they must be contextualized for the topic of our research. A single small Japanese study conducted in healthy children suggests that the lower the age, the higher the antioxidant capacity and oxidative stress level [23]. In the setting of IBD, a longer disease duration can affect the pro- and antioxidant mechanisms as a consequence of the accumulating inflammation and/or the increasing pharmacological load. Currently, the drug availability for IBD children is still limited compared to adults, particularly for biologics and new oral molecules [56]. As emerged from our data, a high proportion of IBD children are on immunomodulators (e.g., azathioprine), and the use of biologics is almost limited to anti-TNF alpha. The pharmacological effect of azathioprine is known to contribute to oxidative stress [57], while newer and more potent anti-inflammatory drugs might have a different impact on the oxidative burden in IBD. Interestingly, in the total cohort, the only drug associated with an improved antioxidant capacity (increased FRAP) was mesalazine, which was largely used in adults rather than in children. Differences in the length of disease, consequent exposure to the inflammatory burden, and the pharmacological load could explain antioxidant capacities modified by age; however, to further explore these aspects, longitudinal studies would be necessary.

Disease activity (measured by fecal calprotectin) was inversely correlated to the total antioxidant capacity in the entire cohort and directly correlated to the levels of AOPPs only in IBD children. Previous studies revealed direct correlations between disease activity in IBD and the total antioxidant ability, and the results of meta-analysis confirm this correlation [10]. In previous reports, the definition of active disease was mainly based on clinical or endoscopic validated scores. Ours is one of the first studies to include fecal calprotectin as a marker of disease activity/inflammation and correlate it with oxidative markers. In a previous study in IBD adults, a direct correlation was found between fecal calprotectin and the activity of antioxidant enzymes (GPX and CAT), but not with other markers (TAC and MDA) [58]. Calprotectin, a protein mainly released by neutrophils, has been involved in the inhibition of neutrophil oxidative metabolism [59]. It has already been suggested that calprotectin may have a role mediated by the production of ROS or an antioxidant role amplifying antioxidant protection [60].

Unexpectedly, levels of vitamin C (generally recognized as an endogenous antioxidant) were found to be positively associated with levels of AOPPs (a marker of oxidative damage) (Table 5). Circulating vitamin C levels may reflect not only antioxidant capacity but also dietary intake, supplementation, or compensatory responses to increased oxidative stress. In this context, higher vitamin C concentrations could be observed in individuals experiencing greater oxidative burden, which may also be associated with elevated levels of protein oxidation markers such as AOPPs. Therefore, the positive association observed in our study may reflect a complex relationship between oxidative stress and antioxidant status rather than a direct causal effect. We should also acknowledge a potential analytical interference between reducing substances (such as ascorbic acid) and AOPP assays based on chloramine-T absorbance, such as our method [34]. However, the circulating vitamin C concentrations observed in our cohort were within the physiological range and substantially lower than levels typically associated with significant in vitro interference.

Lastly, the quantile regression analysis for the total cohort (Table 7, Table 8 and Table 9) showed a negative association of the female gender and the diagnosis of UC/IBD-U with the total antioxidant capacity and a positive association of AOPPs with the diagnosis of CD. Sex hormones, particularly estrogens, have been shown to impact many pro- and anti-oxidative mechanisms [57]. Previous studies have already depicted a variable oxidative imbalance, according to the IBD subtype [61]. Differences can be influenced by disease location, with a consequent different nutrient absorption and severity of inflammation. CD subjects have shown a higher rate of lipid oxidation and lower carbohydrate oxidation compared to UC patients and healthy individuals [62]. Raised AOPP levels have been reported in both CD and UC studies [46,49,50,51,52,53], with a single adult study comparing protein oxidation products, expressed as the concentration of IMA (ischemia-modified albumin) and revealing significantly higher values in UC compared to CD [63].

In summary, our study, the first to explore oxidative markers in children with IBD in comparison to adults, shows a reduced total antioxidant capacity and increased oxidation products in IBD children in comparison to adults and healthy controls. Many of these alterations appear more evident in younger patients, female patients, and those with clinical activity. We assessed oxidative status by validated, reproducible, and easy-to-use methods. Furthermore, we explored many conflicting parameters of antioxidant mechanisms and oxidative damage to best elucidate the global oxidative status.

We must underline that the generalization of our data is limited by the small number of patients enrolled, the heterogeneity of the cohort (including CD, UC, and IBD-U patients under different treatments), and the low percentage of patients with active disease. Remission was defined based on clinical indices and not endoscopic parameters, so we must recognize this as a limitation. However, these indices are commonly used in clinical practice as non-invasive surrogates to define disease activity, particularly in a pediatric setting. We recognize the limitations of the selected control groups (including medical staff for adults and healthy children undergoing a pediatric check-up), which may not be entirely representative of the general population. We also acknowledge that residual confounding factors related to lifestyle cannot be fully excluded.

In addition, the analysis included multiple biomarkers and subgroup comparisons, and the multiple models were derived through variable selection in a relatively modest sample. Therefore, the stability of the selected models cannot be guaranteed, and some associations may be sample-dependent. These findings should therefore be interpreted with caution, particularly in the smaller subgroups. The observational nature of our data, although representative of a real-life cohort, could be better validated with a prospective design.

## 5. Conclusions

In summary, this exploratory study found reduced global antioxidant capacity, particularly FRAP, in children with IBD, and an association between AOPPs and disease activity in the pediatric subgroup. These findings suggest age-, disease-, treatment-, and nutritional-status-related differences in oxidative balance, but require confirmation in larger, longitudinal, population-based cohorts before oxidative-stress markers can be used for prediction, monitoring, or therapeutic targeting.

## Figures and Tables

**Table 1:** Clinical and demographical characteristics of the total population.

Characteristics	pIBD (n = 41)	pControls (n = 34)	*p*	aIBD (n = 41)	aControls (n = 39)	*p*	pIBD vs. aIBD *p*
Age, years	13.3 (12.3; 14.5)	12.0 (10.0; 14.0)	0.035 ^a^	30.5 (24.7–42.9)	32.0 (27.0–41)	0.343 ^a^	-
Age at diagnosis, years	10.7 (3.4; 12.7)	-		23 (16.8–31.0)	-	-	-
Gender, female	24 (58.5)	17 (50)	0.613 ^b^	22 (53.8)	21 (53.7)	0.999 ^b^	0.090 ^b^
Type of disease:						-	0.058 ^b^
Crohn	22 (53.7)	-	-	21 (51.2)	-		
UC/IBD-U	19 (46.3)	-	-	20 (48.3)	-		
Active disease Remission	13 (31.7) 28 (68.3)	-	-	14 (34.2) 27 (65.8)	-	-	0.999 ^b^
Extra-intestinal manifestations	4 (9.8)	-	-	9 (22.0)	-	-	0.226 ^c^
Previous surgery	2 (4.9)	-	-	7 (17.1)	-	-	0.155 ^c^
Immunomodulators	27 (65.9)	-	-	7 (17.1)	-	-	**<0.001** ^b^
Steroids	1 (2.4)	-	-	3 (7.7)	-	-	0.233 ^c^
Mesalazine	14 (34.1)	-	-	23 (59.0)	-	-	0.061 ^b^
Biologics	25 (61.0)	-	-	31 (75.6)	-	-	0.235 ^b^
Infliximab	23 (56.1)			16 (39.0)			
Adalimumab	2 (4.9)			7 (17.1)			
Vedolizumab	-			6 (14.6)			
Ustekinumab	-			1 (2.4)			
Golimumab	-			1 (2.4)			
Probiotics	2 (4.9)	-	-	5 (12.2)	-	-	0.432 ^c^
Vitamin D supplements	13 (31.7)	-	-	9 (22.0)	-	-	0.455 ^b^
Exclusive enteral nutrition	7 (17.1)	-	-	2 (4.9)	-	-	0.155 ^c^
Special exclusion diet	15 (36.6)	-	-	8 (19.5)	-	-	**0.011** ^b^
BMI, kg/m^2^	18.0 (16.6; 20.2)	18.6 (15.5; 21.8)	0.866 ^a^	23.3 (21.2; 25.6)	22.8 (21.0; 25.8)	0.999 ^b^	**<0.001** ^a^
Nutritional status			0.608 ^c^			-	**0.010** ^c^
Mild and severe underweight	12 (29.3)	6 (17.6)		5 (12.2)	0 (0)		
Normal weight	24 (58.5)	22 (64.7)		19 (46.3)	3 (7.7)		
Overweight + Obese	5 (12.2)	4 (11.8)		13 (31.7)	1 (2.6)		
Not available	-	2 (5.9)		4 (9.8)	35 (89.7)		
Fecal calprotectin, mg/kg	151.5 (43; 500)	19 (15; 32.3)	**<0.001** ^a^	135 (45–333)	20 (15; 45)	**<0.001** ^a^	0.817 ^a^

Age, age at diagnosis, BMI, fecal calprotectin are expressed as median and interquartile range (IQR). The other variables are reported as number of subjects (%). *p*: *p*-values: ^a^ Wilcoxon rank-sum test, ^b^ Chi-square test, ^c^ Fisher’s exact test. UC: Ulcerative Colitis; IBD-U: unclassified Inflammatory Bowel Disease. Significant differences are reported in bold.

**Table 2:** Oxidative stress parameters in the total population.

	pIBD (n = 41)	pControls (n = 34)	*p*	aIBD (n = 41)	aControls (n = 39)	*p*	pIBD vs. aIBD *p*
FRAP (mcmol/eq Fe^2+^)	194 (178.6; 214.0)	239.4 (210.8; 264.5)	**<0.001** ^a^	259.9 (225.4; 295.0)	247.9 (220; 295.6)	0.740 ^a^	**<0.001** ^a^
TBARSs (mcmol/MDA)	8.9 (7.9; 11.0)	9.2 (7.7; 10.4)	0.569 ^a^	10.4 (8.8; 13.0)	10.1 (8.7; 11.7)	0.310 ^a^	**0.023** ^a^
AOPPs (mcmol/gr plasm prot)	6.39 (4.7; 7.0)	5.0 (4.5; 6.0)	**0.011** ^a^	5.8 (4.7; 6.6)	5.4 (4.4; 6.8)	0.392 ^a^	0.378 ^a^
ROS	40.8 (28.9; 94.5)	35.6 (27.0; 51.7)	0.119 ^a^	38.3 (26.3; 63.9)	35.6 (26.0; 45.0)	0.351 ^a^	0.3017 ^a^
Total GSH	19.3 (14.6; 22.8)	29.1 (13.3; 42.3)	**0.033** ^a^	13.6 (9.9; 20.8)	14.7 (9.6; 23.3)	0.585 ^a^	**0.020** ^a^
GPX, nmol/mL/mg	50.8 (33.5; 58.5)	37.3 (22.7; 52.9)	0.074 ^a^	39.3 (26.7; 56.0)	37.6 (27.8; 67.5)	0.740 ^a^	0.155 ^a^
GST, nmol/mL/mg	7.8 (5.2; 10.6)	8.9 (5.6; 12.3)	0.367 ^a^	9.6 (6.9; 12.9)	7.9 (6.3; 10.4)	0.137 ^a^	0.071 ^a^
GR, nmol/mL/mg	14.2 (9.2; 18.2)	13.9 (8.4; 22.3)	0.337 ^a^	12.5 (7.6; 26.6)	15.7 (12.2; 21.5)	0.289 ^a^	0.904 ^a^
CAT, nmol/mL/mg	83.7 (52.8; 132.0)	54.8 (32.8; 74.7)	**0.010** ^a^	109.8 (61.0; 188.8)	70.2 (46.6; 106.9)	0.032 ^a^	0.356 ^a^
SOD, U/mg	5.2 (3.9; 6.3)	3.6 (2.6; 5.5)	**0.039** ^a^	5.2 (3.9; 7.7)	4.4 (2.9; 6.3)	0.130 ^a^	0.651 ^a^

All variables are expressed as median and interquartile range (IQR).; *p*: *p*-values: ^a^ Wilcoxon rank-sum test, FRAP: ferric-reducing activity of plasma; TBARSs: thiobarbituric acid reactive substances; AOPPs: advanced oxidative protein products; ROS: reactive oxygen species; GSH: glutathione; GPX: glutathione peroxidase; GST: glutathione S-transferase; GR: glutathione reductase; CAT: catalase; SOD: superoxide dismutase. Significant differences are reported in bold.

**Table 3:** Oxidative markers (FRAP, AOPPs, TBARSs) in active and remission status of disease.

	pIBDR (n = 28)	pIBDA (n = 13)	*p*	aIBDR (n = 27)	aIBDA (n = 14)	*p*	tIBDR (n = 55)	tIBDA (n = 27)	*p*
**FRAP** **(mcmol/eq Fe^2+^)**	193 (174; 214)	195 (184; 226)	0.394 ^a^	267 (217; 304)	250.5 (224.3; 276.8)	0.418 ^a^	214 (186; 267)	228 (192; 263)	0.718 ^a^
**AOPPs** **(mcmol/gr plasm prot)**	5.9 (4.4; 6.9)	6.7 (6.4; 7.65)	**0.014** ^a^	5.9 (4.7; 6.7)	5.6 (4.7; 6.6)	0.812 ^a^	5.9 (4.5; 6.8)	6.5 (5.2; 6.9)	0.088 ^a^
**TBARSs** **(mcmol/MDA)**	9.6 (8.3; 10.8)	8.4 (7.3; 13.4)	0.314 ^a^	10.8 (8.8; 16.2)	9.6 (8.5; 11.3)	0.254 ^a^	10.1 (8.7; 12.5)	9 (8.1; 11.9)	0.216 ^a^

All variables are expressed as median and interquartile range (IQR). *p*: *p*-values: ^a^ Wilcoxon rank-sum test. Disease activity was defined based on clinical scores (w-PCDAI and PUCAI for children with CD and UC/IBD-U, respectively; Harvey–Bradshaw index and Mayo score for adults with UC/IBD-U and CD, respectively). pIBDR: pediatric Inflammatory Bowel Disease in remission, pIBDA: pediatric Inflammatory Bowel Disease with active disease, aIBDR: adult Inflammatory Bowel Disease in remission, aIBDA: adult Inflammatory Bowel Disease with active disease, tIBDR: total IBD population in remission, tIBDA: total IBD population with active disease; FRAP: ferric-reducing activity of plasma, AOPPs: advanced oxidative protein products; TBARSs: thiobarbituric acid reactive substances; MDA: malondialdehyde. Significant differences are reported in bold.

**Table 4:** Association between patient characteristics and FRAP: results of quantile regression in IBD children vs. controls (n = 66).

	Antioxidant Capacity (FRAP)	
Variables	Coeff.	95% CI
Disease (Yes vs. No)	**−53.033**	**−64.047; −42.19**
Age, years	**7.316**	**1.7; 10.377**
Gender (male vs. female)	**10.755**	**7.614; 21.214**
Vitamin A	**0.52**	**0.033; 1.347**

Coeff: Change in the median of the dependent variable for a one-unit change in the independent variable; 95% CI: 95% confidence interval; FRAP: ferric-reducing activity of plasma. Significant differences are reported in bold.

**Table 5:** Association between patient characteristics and AOPPs: results of quantile regression in IBD children vs. controls (n = 43).

	Oxidative Products (AOPPs)	
Variables	Coeff.	95% CI
Disease (Yes vs. No)	0.166	−1.323; 0.694
Age, years	**0.136**	**0.12; 0.428**
Fecal calprotectin, mg/kg	**0.002**	**0; 0.005**
Vitamin A	−0.041	−0.062; 0.018
Vitamin C	**0.052**	**0.024; 0.258**

Coeff: Change in the median of the dependent variable for a one-unit change in the independent variable; 95% CI: 95% confidence interval; AOPPs: advanced oxidative protein products. Significant differences are reported in bold.

**Table 6:** Association between patient characteristics and oxidative stress (SOD: superoxide dismutase): results of quantile regression (n = 41).

	Antioxidant Activity (SOD)	
Variables	Coeff.	95% CI
Disease (Yes vs. No)	0.364	−0.303; 2.23
Fecal calprotectin, mg/kg	**0.003**	**0.001; 0.005**
Vitamin A	−0.019	−0.05; 0.025
Vitamin C	**0.198**	**0.02; 0.316**

Coeff: Change in the median of the dependent variable for a one-unit change in the independent variable; 95% CI: 95% confidence interval; SOD: superoxide dismutase. Significant differences are reported in bold.

**Table 7:** Association between patient characteristics and FRAP: results of quantile regression in IBD children vs. IBD adults (n = 73).

	Antioxidant Capacity (FRAP)	
Variables	Coeff.	95% CI
Gender (male vs. female)	**8.12**	**3.92; 28.515**
Adults vs. children	**30.673**	**13.65; 53.545**
UC/IBD-U vs. Crohn	**−23.456**	**−40.22; −12.532**
Biologics (yes vs. no)	−4.17	−16.13; 16.078
Immunomodulators (yes vs. no)	−18.476	−29.3; 8.554
Mesalazine (yes vs. no)	**27.932**	**8.39; 41.479**
Fecal calprotectin, mg/kg	**−0.037**	**−0.07; −0.015**
Nutritional status:		
Underweight vs. normal weight	−6.254	−24.75; 26.176
Overweight vs. normal weight	**32.733**	**15.5; 66.925**

Coeff: Change in the median of the dependent variable for a one-unit change in the independent variable; 95% CI: 95% confidence interval; FRAP: ferric-reducing activity of plasma. Significant differences are reported in bold.

**Table 8:** Association between patient characteristics and AOPPs: results of quantile regression in IBD children vs. IBD adults (n = 73).

	Oxidative Products (AOPPs)	
Variables	Coeff.	95% CI
Gender (male vs. female)	0.14	−0.37; 1.005
Adults vs. children	−0.094	−1.02; 0.301
UC/IBD-U vs. Crohn	**−0.6**	**−1.6; −0.367**
Biologics (yes vs. no)	0.275	−0.76; 0.749
Immunomodulators (yes vs. no)	0.241	−0.51; 0.959
Mesalazine (yes vs. no)	0.202	−0.18; 0.982
Fecal calprotectin, mg/kg	**0.001**	**0; 0.003**
Nutritional status:		
Underweight vs. normal weight	−0.831	−1.13; 0.308
Overweight vs. normal weight	−0.553	−1.19; 1.153

Coeff: Change in the median of the dependent variable for a one-unit change in the independent variable; 95% CI: 95% confidence interval; AOPPs: advanced oxidative protein products. Significant differences are reported in bold.

**Table 9:** Association between patient characteristics and oxidative stress (SOD: superoxide dismutase): results of quantile regression (n = 73).

	Antioxidant Activity (SOD)	
Variables	Coeff.	95% CI
Gender (male vs. female)	−0.094	−0.531; 1.232
Adults vs. children	0.359	−0.383; 1.856
**UC/IBDU vs. Crohn**	**1.657**	**0.500; 2.183**
Biologics (yes vs. no)	−0.242	−1.073; 0.85
Immunomodulators (yes vs. no)	−0.648	−0.957; 0.536
Mesalazine (yes vs. no)	−0.334	−1.195; 1.22
Fecal calprotectin, mg/kg	0.002	−0.001; 0.002
Nutritional status:		
Underweight vs. normal weight	−0.349	−1.294; 1.574
Overweight vs. normal weight	−1.299	−3.112; 0.26

Coeff: Change in the median of the dependent variable for a one-unit change in the independent variable; 95% CI: 95% confidence interval; SOD: superoxide dismutase. Significant differences are reported in bold.

## Data Availability

The data are available from the corresponding author upon reasonable request.

## Supplementary Materials

The following supporting information can be downloaded at: https://www.mdpi.com/article/10.3390/nu18091458/s1. Table S1: Blood tests, vitamins, and micronutrient values in children and adults with IBD. Table S2a: Association between patient characteristics and FRAP: results of quantile regression IBD adults vs. controls (n = 81). Table S2b: Association between patient characteristics and AOPP: results of quantile regression IBD adults vs. controls (n = 80). Table S2c: Association between patient characteristics and oxidative stress (SOD: superoxide dismutase): results of quantile regression (n = 80).

## Abbreviations

The following abbreviations are used in this manuscript:

IBD	Inflammatory Bowel Disease
CD	Crohn’s disease
UC	Ulcerative Colitis
IBD-U	IBD-unclassified
OS	oxidative stress
ROS	reactive oxygen species
RNS	reactive nitrogen species
SOD	superoxide dismutase
CAT	catalase
GPX	glutathione peroxidase
PON1	paraoxonase
GSH	glutathione
FRAP	ferric-reducing activity of plasma
TBARSs	thiobarbituric acid reactive substances
AOPPs	advanced oxidation protein products
TAC	total antioxidant capacity
