# Supplementary material for: The Oxidative Stress Imbalance in Children and Adults with IBD and Associated Factors
Source: Nutrients. 2026 May 1;18(9):1458. doi: 10.3390/nu18091458 (PMC13164794; doi:10.3390/nu18091458)
Supplement: Supplementary file 1 [file nutrients-18-01458-s001.zip › nutrients-4285025-supplementary.pdf]

Supplementary tables

Supplementary table S1. Blood tests, vitamins, and micronutrient values in children and adults with IBD.

	**pIBD (n = 41)**	**pControls (n = 34)**	***p***	**aIBD (n=41)**	**pIBD vs aIBD *p***
Hb, g/dl	12.9 (12.1-13.8)	13.3 (12.9-13.7)	0.118 [a]	13.4 (12.6-14.0)	0.233[a]
PLTs, n*10^3/mmc	298 (243-364)	253.5 (217.75-317.25)	**0.039** [a]	249.5 (216.5-303.75)	**0.022**[a]
ESR, mm/h	10.0 (6-19)	4.0 (2-11)	**0.032** [a]	9.5(2.8-12.8)	0.454[a]
CRP, mg/dl	0.3 (0.3-0.3)	0.3 (0.3-0.3)	0.234 [a]	0.3(0.3-0.6)	0.84[a]
Albumin, g/dL	4.0 (3.9; 4.3)	4.3 (4.2; 4.5)	**0.001** [a]	4.1(3.9-4.3)	0.498[a]
Urea, mmol/dL	24 (22; 28)	28 (24; 32)	0.056 [a]	32.5(23.0-40.0)	0.064[a]
Creatinine, mg/dL	0.5 (0.4-0.6)	0.5 (0.5; 0.6)	0.224 [a]	0.8 (0.7-0.8)	**<0.001**[a]
Glycemia, mg/dL	80.0 (78-85)	83.0 (78; 87)	0.392 [a]	89.0 (79-93)	0.074[a]
ALT, U/l	20.5 (16; 28)	21.0 (18; 26)	0.334 [a]	25.0 (17-32)	0.547[a]
Zinc, µg/dL	98.0 (76-119)	108.0 (88; 146)	0.193 [a]	84.0 (69-103)	0.367[a]
Ferritin, ng/mL	30.0 (16; 52)	31.0 (25; 45)	0.605 [a]	27.0 (14-75)	0.915[a]
Vitamin A, µg/dL	40.0 (32; 44)	38.0 (33; 45)	0.802 [a]	41.0 (38; 44)	0.408[a]
Vitamin E, µg/dL	1102.0 (968; 1252)	1164.0 (916; 1390)	0.995 [a]	1156.0 (1089; 1419)	0.483[a]
Vitamin C, mg/L	7.4 (5.2; 9.9)	8.0 (4.4; 9.4)	0.994 [a]	7.4 (2.9; 10.0)	0.641[a]
Vitamin D, ng/mL	24.3 (18.6; 29.9)	20.8 (16.5; 25.9)	0.163 [a]	21.1 (17; 35)	0.237[a]
Total Cholesterol, mg/dL	151.0 (120.8; 166.5)	154.0 (135.5; 187.2)	0.174 [a]	170.0 (131; 196)	0.208[a]
Cholesterol HDL, mg/dL	54.5 (45.5; 67.3)	51.0 (44.5; 65.0)	0.738 [a]	53.0 (47; 70)	0.714[a]
Triglycerides, mg/dL	66.0 (51.0; 95.5)	63.0 (50.0; 101.5)	0.762 [a]	82.0 (44; 96)	0.517[a]

All variables are expressed as median and InterQuartile Range (IQR). p: p-values: [a]Wilcoxon sum-rank test.
pIBD: pediatric Inflammatory Bowel Disease, aIBD: adult Inflammatory Bowel Disease. Hb: hemoglobin, PLTs: platelets, ESR: erythrocyte sedimentation rate, CRP: C-reactive protein, ALT: alanine amino transferase.

Supplementary table S2a. Association between patient characteristics and FRAP: results of quantile regression IBD adults vs. controls (n = 81).

	**Antioxidant capacity (FRAP)**	
**Variables**	**Coeff.**	**95%CI**
Disease (yes vs no)	0.737	-20.22; 25.337
Gender (Male vs Female)	0.324	-1.09; 1.317
Age (years)	47.542	32.83; 69.623

95%CI: 95% Confidence Interval

Supplementary table S2b. Association between patient characteristics and AOPP: results of quantile regression IBD adults vs. controls (n = 80).

	Oxidative products (AOPP)	
**Variables**	**Coeff.**	**95%CI**
Disease (yes vs no)	0.018	-0.69; 1.264
Gender (Male vs Female)	-0.006	-0.06; 0.019
Age (years)	0.462	-0.54; 1.486

95%CI: 95% Confidence Interval

Supplementary table S2c. Association between patient characteristics and oxidative stress (SOD: superoxide dismutase): results of quantile regression (n = 80)

	Atioxidant activity (SOD)	
**Variables**	**Coeff.**	**95%CI**
**Disease (yes vs no)**	**0.951**	0.33; 2.342
Gender (Male vs Female)	-0.005	-0.04; 0.078
**Age (years)**	**-1.141**	-2.12; -0.185

95%CI: 95% Confidence Interval

FRAP: ferric reducing activity of plasma; AOPP: advanced oxidative protein products; SOD: Superoxide Dismutase.
